# Factors associated with bacteraemia due to multidrug-resistant organisms among bacteraemic patients with multidrug-resistant organism carriage: a case control study

**DOI:** 10.1186/s13756-018-0412-3

**Published:** 2018-09-29

**Authors:** Hélène Mascitti, Clara Duran, Elisabeth-Marie Nemo, Frédérique Bouchand, Ruxandra Câlin, Alexis Descatha, Jean-Louis Gaillard, Christine Lawrence, Benjamin Davido, François Barbier, Aurélien Dinh

**Affiliations:** 1 Infectious disease unit, Raymond Poincaré University Hospital, AP-HP, Versailles Saint-Quentin University, 104 Bd R. Poincaré, 92380 Garches, France; 2 Pharmacy department, Raymond Poincaré University Hospital, AP-HP, Versailles Saint-Quentin University, 104 Bd R. Poincaré, 92380 Garches, France; 3 Microbiological laboratory, Raymond Poincaré University Hospital, AP-HP, Versailles Saint-Quentin University, 104 Bd R. Poincaré, 92380 Garches, France; 4 Intensive care unit, Orléans Hospital, 14 Avenue de l’Hôpital, 45067 Orléans, France

**Keywords:** Multidrug-resistant organism, Antimicrobial, Bacteraemia, Carriage

## Abstract

**Background:**

Infections caused by multidrug-resistant organisms (MDRO) are emerging worldwide. Physicians are increasingly faced with the question of whether patients need empiric antibiotic treatment covering these pathogens. This question is especially essential among MDRO carriers. We aim to determine the occurrence of MDRO bacteraemia among bacteraemic patients colonized with MDRO, and the associated factors with MDRO bacteraemia among this population.

**Methods:**

We performed a retrospective monocentric study among MDRO carriers hospitalized with bacteraemia between January 2013 and August 2016 in a French hospital. We compared characteristics of patients with MDRO and non-MDRO bacteraemia.

**Results:**

Overall, 368 episodes of bacteraemia were reviewed; 98/368 (26.6%) occurred among MDRO carriers.

Main colonizing bacteria were extended-spectrum beta-lactamase (ESBL)-producing *Escherichia coli* (40/98; 40.8%), ESBL-producing *Klebsiella pneumoniae* (35/98; 35.7%); methicillin-resistant *Staphylococcus aureus* (26/98; 26.5%) and multidrug-resistant *Pseudomonas aeruginosa* (PA) (12/98; 12.2%).

There was no significant difference considering population with MDRO bacteraemia vs. non-MDRO bacteraemia, except for immunosuppression [OR 2.86; *p* = 0.0207], severity of the episode [OR 3.13; *p* = 0.0232], carriage of PA [OR 5.24; *p* = 0.0395], and hospital-acquired infection [OR 2.49; *p* = 0.034].

In the multivariate analysis, factors significantly associated with MDRO bacteraemia among colonized patient were only immunosuppression [OR = 2.96; *p* = 0.0354] and the hospital-acquired origin of bacteraemia [OR = 2.62; *p* = 0.0427].

**Conclusions:**

According to our study, occurrence of bacteraemia due to MDRO among MDRO carriers was high. Factors associated with MDRO bacteraemia were severity of the episode and hospital-acquired origin of the bacteraemia. Thus, during bacteraemia among patients colonized with MDRO, if such characteristics are present, broad-spectrum antimicrobial treatment is recommended.

## Background

There is currently an epidemiologic dramatic increase of multidrug-resistant organisms (MDRO) [1–5].

Infections caused by MDRO have been associated with severe adverse clinical outcomes, leading to increased mortality, prolonged hospital stay, and increased costs, mostly because of delayed effective therapy [6–9]. This dramatic spread takes place in both the community and hospital setting.

However, colonization and infection due to MDRO should be differentiated.

At this time, colonization with MDRO among patients is more frequent than infection.

But colonization with MDRO is a risk factor for infections due to MDRO, especially in transplanted patients and in intensive care unit [10–12].

If sepsis or sepsis-mimicking events occur among MDRO carriers, effective probabilistic broad-spectrum antibiotics are often prescribed in common practice [13]. Consequently, broad-spectrum antimicrobial treatments are increasingly used as empiric therapy among colonized patients. It could lead to unnecessary antibiotic exposure and selective pressure, creating more bacterial resistance.

This vicious circle is worryingly contributing to a rapid international dissemination of MDRO [14–16].

Physicians should therefore consider a prudent use of broad-spectrum antibiotics to limit new emergence of MDRO.

This requires updated studies to identify current risk factors for MDRO infection among MDRO carriers.

The primary objective of our study was to determine the occurrence of MDRO bacteraemia among bacteraemic patients colonized with MDRO, and which associated factors are predictive of bacteraemia due to MDRO among this population.

## Methods

### Settings and design

We performed a retrospective monocentric study among MDRO carriers (from any site: urine, respiratory, digestive, cutaneous), hospitalized with bacteraemia between January 2013 and August 2016 in our teaching hospital, according to STROBE statement [17]. We compared characteristics of patients with MDRO and non-MDRO bacteraemia.

Our university hospital is a disability referral centre for neurological impairment, including spinal cord injured patients. These patients are subject to high antimicrobial exposure because they might have a high rate of infections, especially urinary tract infections; they are also at increased risk of infection with multidrug-resistant bacteria [18–20]. The hospital has 255 acute-care facility beds (including 28 beds of intensive care unit) and 108 for rehabilitation, with around 8400 admissions annually. Average hospital stays are 6.9 days for acute care and 36.5 days for rehabilitation.

An active surveillance policy for MDRO carriage among high-risk patients is implemented: nasal swab for methicillin-resistant *Staphylococcus aureus* (MRSA), and rectal swab for Gram negative resistant bacteria and vancomycin-resistant enterococci.

Systematic screening is performed at hospital admission for all patients coming from acute or long-term care facilities, and for community patients previously known as carriers.

Moreover, weekly screening is performed in our intensive care and surgery departments.

All hospitalized patients with positive blood cultures for bacteria were identified from the microbiology laboratory database, and microbiological data was obtained and reviewed. Patients with MDRO carriage (at least one site) during the last 3 months until day of sepsis were included.

Medical charts were reviewed using a standardized data set to collect: demographic characteristics (age, sex, comorbidities, risk factors, etc); clinical, biological, and microbiological data (clinical and severity signs, laboratory tests, organisms identified), and outcomes of each episode.

Blood cultures were performed using aerobic and anaerobic blood culture vials incubated in a Bactec FX instrument (Bactec Ped+ and Lytic/10 Anaerobic/F, BD Diagnostics, Le Pont de Claix, France). The positive blood culture vials were subcultured on blood and chocolate Polyvitex agar plates. All isolates were then identified using MALDI-TOF mass spectrometry (Maldi Biotyper 3.0, Bruker Daltonique, Marnes la Vallée, France).

Antimicrobial susceptibility testing was carried out using the agar disk diffusion method (Bio-Rad) or an automated broth microdilution method (Phoenix, BD Diagnostics, Oxford, UK). The breakpoints used were those defined by the French Committee for Antimicrobial Susceptibility Testing (http://www.sfm-microbiologie.org/UserFiles/files/casfm/CASFM%20V1_0%20FEV_2018.pdf).

### Definitions

**Bacteraemia** was defined as the association of at least one positive blood culture and a prescription of a systemic antibiotic treatment to treat bacteraemia. For common skin contaminants, such as coagulase-negative staphylococci or *Corynebacterium* sp., at least two different sets of blood cultures were required.

**Polymicrobial bacteraemia** was defined as having more than one organism found in the same bacteraemic episode.

**MDRO status** was determined for the *Enterobacteriaceae* group, *Acinetobacter* sp., *Pseudomonas aeruginosa*, and *Enterococcus* sp. as acquired non-susceptibility to at least one agent in three or more antimicrobial categories; for *Staphylococcus aureus* as resistance to methicillin [21].

**High zone of prevalence of MDRO** were southern Europe (Spain, Italy, Greece), North Africa and Asia according to European Centre for Disease Prevention and Control (ECDC) data (https://www.ecdc.europa.eu/en/home).

**Hospital-acquired infection** was determined as clinical signs of infection or infection arise at least 48 h after hospital admission.

**Prior colonization** was defined as isolation of MDRO from any site without any clinical signs of inflammation or sepsis, and antibiotic therapies targeting these MDRO, within a designated period of 3 months before the day of bacteraemia.

**Prior antibiotic use** was defined as the use of at least 1 dose of any antimicrobial treatment in a designated period of 3 months until the day before sepsis.

**Immunosuppression** included the following**:** diabetes mellitus, ongoing neoplasia, hemopathy, HIV, hypogamma globulinemia, immunosuppressive therapy (ie. corticotherapy > 20 mg/d, chemotherapy or immunosuppressive treatment such as cyclophosphamide, azathioprine and cyclosporine).

**Primary site of infection** were clinically suspected (by the physician in charge or reported on the medical chart) or bacteriologically documented with the same bacterial identification as that in the blood culture. Primary sites were categorized as urinary tract infection, catheter line-associated bacteraemia, osteoarticular infection, pulmonary tract infection, skin and soft tissue infection, intra-abdominal infection, and unknown when no primary site had been identified.

**Severity** was defined as the requirement of at least one of the following criteria: volume expansion, assisted (mechanical) ventilation, vasopressor requirement, and intensive care unit (ICU) admission during the episode.

**Cure** was defined as the absence of clinical and biological signs of infection at 1 month after end of antimicrobial treatment or at hospital discharge without any additional antimicrobial treatment.

**Mortality** was defined as dead status before 30 days after the end of antimicrobial treatment.

### Statistical analysis

All continuous variables are presented as mean and standard deviation, and the categorical variables are presented as frequencies. Correlations between risk factors and MDRO bacteraemia among patients colonized with MDRO were determined by Student’s t-test for continuous variables and the Pearson’s χ2 test for categorical variables.

Univariate analysis and multivariate analysis were performed. Variable for multivariate analysis were all associated risks that had a *p*-value ≤ 0.05 and sex in the univariate analysis.

The relative risks of MDROs bacteraemia were estimated by calculating the adjusted odds ratios (OR) and corresponding 95% confidence intervals (CI).

All reported probability values (*P*-values) were based on two-sided tests, and a *P*-value of 0.05 was considered statistically significant. All analyses were performed using the Statistical Package for Social Science (SPSS) version 17.0 (SPSS, Chicago, IL, USA).

## Results

During the study period, a mean of 198 ± 54 screening per month was performed, and mean positive results for MDRO per patient was 23 ± 5%, with 45% of extended-spectrum beta-lactamase (ESBL)-producers isolates.

**Fig. 1:**
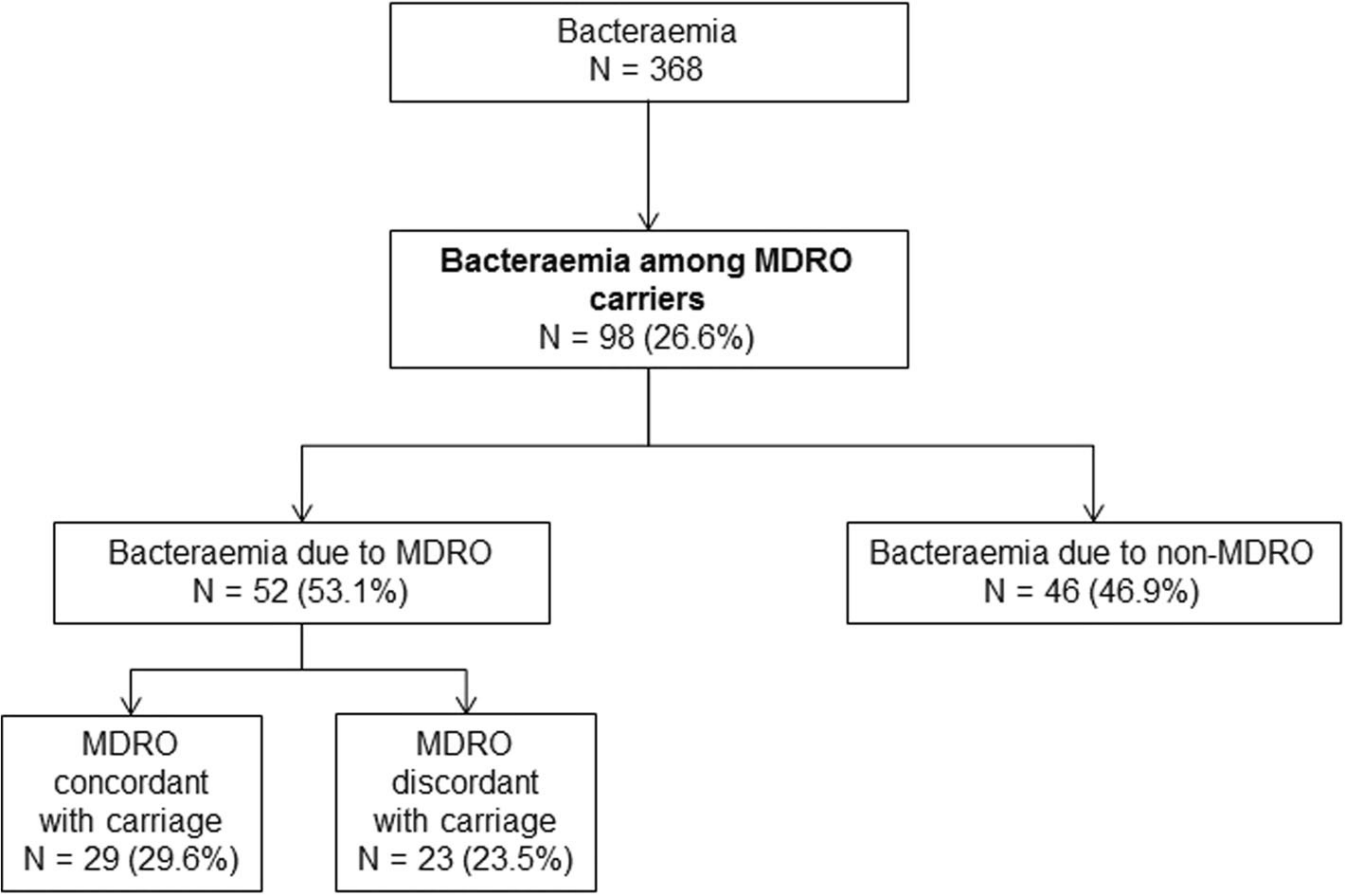
Study flow chart

In total, 368 episodes of bacteraemia were reviewed; 98 (26.6%) occurred among 77 MDRO carriers (Fig. 1). Eight bacteraemia episodes were plurimicrobial.

Considering the 98 episodes of bacteraemia among MDRO carriers, mean age was 55.8 years old, and sex ratio was 1.65. Prior antimicrobial treatment in the last 3 months occurred in 66 (67.3%) cases, 42 (42.9%) patients had an indwelling catheter, 33 (33.7%) were immunosuppressed, and 55 (56.1%) were considered as severe.

Main primary site of infections were urinary tract infections (25; 25.5%) and catheter-line associated infections (25; 25.5%); 12 (12.2%) patients presented primary bacteraemia.

Bacteraemia were hospital-acquired in 62 (63.3%) cases.

The rate of bacteraemia due to MDRO was 53.1% (*n* = 52) (Table 1). Among them, 41 (78.8%) episodes were due to multidrug-resistant *Enterobacteriaceae*, of which 22 (42.3%) were due to ESBL *Enterobacteriaceae*.

**Table 1:** Main characteristics of multidrug-resistant organism carriers with bacteraemia

Variable	Non MDR bacteraemia (*n* = 46)	MDR bacteraemia (*n* = 52)	Odds Ratio	*P* value
Sex (male)	30 (65.2%)	31 (59.6%)	0.70 [0.35; 1.79]	0.5683
Recent (< 3 months) trip in zone with high MDRO prevalence^a^	6 (13.0%)	3 (5.8%)	0.41 [0.10; 1.74]	0.2250
Prior antimicrobial treatment in last 6 months	28 (60.9%)	38 (73.1%)	1.74 [0.74; 4.09]	0.2004
Urinary indwelling catheter	19 (41.3%)	23 (44.2%)	1.13 [0.51; 2.51]	0.7704
Immunosuppression	10 (21.7%)	23 (44.2%)	2.86 [1.17; 6.95]	*0.0207*
Severity	7 (15.2%)	19 (36.5%)	3.13 [1.17; 8.36]	*0.0232*
Primary site of infection				
UTI	10 (21.7%)	15 (28.8%)	1.46 [0.58; 3.67]	0.4218
Intra abdominal infection	6 (13.0%)	5 (9.6%)	0.71 [0.20; 2.50]	0.5929
Bone and joint infection	4 (8.7%)	0 (0.0%)	0.00 [0.00; I]	0.9710
Respiratory tract infection	2 (4.3%)	6 (11.5%)	2.87 [0.55; 14.98]	0.2113
Skin soft tissue infection	7 (15.2%)	4 (7.7%)	0.46 [0.13; 1.70]	0.2471
Catheter line associated infcetion	10 (21.7%)	15 (28.8%)	1.46 [0.58; 3.67]	0.4218
No primary site of infection	5 (10.9%)	7 (13.5%)	1.28 [0.38; 4.33]	0.6965
Colonization MDR pathogen				
Polymicrobial	15 (32.6%)	17 (32.7%)		
CRE	1 (2.2%)	2 (3.8%)		
ESBL *Escherichia coli*	23 (50.0%)	17 (32.7%)	0.49 [0.22; 1.09]	0.0814
*Klebsiella* spp.	18 (39.1%)	25 (48.1%)	1.44 [0.64; 3.22]	0.3738
ESBL *Klebsiella* spp.	17 (37.0%)	23 (44.2%)		
Carba-R *Klebsiella* spp.	1 (2.2%)	1 (1.9%)		
CASE *Klebsiella* spp.	0 (0.0%)	1 (1.9%)		
*Citrobacter* spp.	1 (2.2%)	2 (3.8%)	1.80 [0.16; 20.53]	0.6360
ESBL *Citrobacter* spp.	1 (2.2%)	2 (3.8%)		
*Enterobacter* spp.	5 (10.9%)	8 (15.4%)	1.63 [0.45; 5.98]	0.4590
ESBL *Enterobacter* spp.	5 (10.9%)	7 (13.5%)		
Carba-R *Enterobacter* spp.	0 (0.0%)	1 (1.9%)		
*Acinetobacter baumanii*	2 (4.3%)	3 (5.8%)	0.43 [0.04; 4.92]	0.4984
ESBL *A. baumanii*	1 (2.2%)	1 (1.9%)		
Carba-R *A. baumanii*	1 (2.2%)	1 (1.9%)		
Cefta-R *A. baumanii*	0 (0.0%)	1 (1.9%)		
*Pseudomonas aeruginosa*	2 (4.3%)	10 (19.2%)	5.24 [1.08; 25.32]	*0.0395*
ESBL *P. aeruginosa*	0 (0.0%)	1 (1.9%)		
Carba-R *P. aeruginosa*	0 (0.0%)	3 (5.8%)		
Cefta-R *P. aeruginosa*	2 (4.3%)	6 (11.5%)		
MRSA	11 (23.9%)	15 (28.8%)	1.29 [0.52; 3.19]	0.5814
VRE	1 (2.2%)	0 (0.0%)	0.88 [0.05; 14.51]	0.9300
Type of infections				
Nosocomial	24 (52.2%)	38 (73.1%)	2.49 [1.07; 5.78]	*0.0340*
Cure rate	39 (84.8%)	44 (84.6%)	0.99 [0.33; 2.97]	0.9817

^a^Geographic area with high incidence of extended-spectrum beta-lactamase-producing bacteria, CRE and VRE: Southern Europe (Spain, Italy, Greece), North Africa and Asia

*Carba-R* Carbapenem-resistant; *CASE* Cephalosporinase-producing; *Cefta-R* Ceftaroline-resistant; *CRE* Carbapenem-resistant Enterobacteriaceae; *ESBL* Extended-spectrum beta-lactamase; *MDR* Multidrug-resistant; *MRSA* Methicillin-resistant Staphylococcus aureus; *VRE* Vancomycin-resistant Enterococci

italicised valued are statistically significant

Overall, main colonizing bacteria were ESBL-producing *Escherichia coli* (EC) (*n* = 40; 40.8%), ESBL-producing *Klebsiella pneumoniae* (KP) (*n* = 35; 35.7%); MRSA (*n* = 26; 26.5%), and *Pseudomonas aeruginosa* (PA) (*n* = 12; 12.2%). Twenty-five patients (for 32 episodes) were carriers of several MDRO. Sites of carriage and microorganisms identified are presented in Table 2.

**Table 2:** Multidrug-resistant organism carriage according to site

	Urinary	Rectal	Respiratory	Cutaneous / Wound
ESBL *Enterobacteriaceae*	30	59	2	13
CRE (NDM + OXA types)	1	2	0	0
CASE *Enterobacteriaceae*	0	1	0	0
ESBL *Pseudomonas aeruginosa*	1	0	0	3
Carba-R *P. aeruginosa*	0	2	0	2
Cefta-R *P. aeruginosa*	0	2	3	0
ESBL *Acinetobacter baumanii*	0	1	0	0
OXA-23 *A. baumanii*	0	2	0	0
Cefta-R *A. baumanii*	0	1	0	0
MRSA	3	0	19	4
VRE	0	1	0	0

*ESBL* Extended-spectrum beta-lactamase; *CRE* Carbapenem-resistant *Enterobacteriaceae*; *CASE* Cephalosporinase; *Carba-R* Carbapenem-resistant; *Cefta-R* Ceftaroline-resistant; *MRSA* Methicillin-resistant *Staphylococcus aureus*; *VRE* Vancomycin-resistant Enterococci

Among carriers with bacteraemia due to MDRO, a discordant identification between carriage and bacteraemia was found in 23 (44.2%) episodes (Table 3).

**Table 3:** Discordant identification between carriage and blood culture

Carriage MDRO	Blood culture MDRO
ESBL *Escherichia coli*	MDR non-ESBL *E. coli*
ESBL *E. coli*	ESBL *K. pneumoniae*
ESBL *E. coli*	ESBL *K. pneumoniae*
CASE *Klebsiella pneumoniae*	MDR *K. pneumoniae*
ESBL *K. pneumoniae*	MDR non-ESBL *E. coli*
ESBL *K. pneumoniae*	MDR *K. pneumoniae*
ESBL *K. pneumoniae*	MDR *Proteus mirabilis*
ESBL *K. pneumoniae*	MDR *Serratia marcescens*
ESBL *K. pneumoniae*	Cefta-R *P. aeruginosa*
Cefta-R *Pseudomonas aeruginosa*	MDR non-ESBL *E. coli*
Cefta-R *P. aeruginosa*	MDR *Enterobacter cloacae*
ESBL *E. cloacae*	MDR *P. mirabilis*
ESBL *Acinetobacter baumanii*	MDR non-ESBL *E. coli*
Cefta-R *A. baumanii*	MDR *P. aeruginosa*
ESBL *Morganella morganii*	MDR Providencia stuartii
MRSA	MDR non-ESBL *E. coli*
MRSA	MDR non-ESBL *E. coli*
MRSA	ESBL *K. pneumoniae*
MRSA	MDR *Enterococcus faecium*
ESBL *E. coli*ESBL *K. pneumoniae*MRSA	MDR non-ESBL *E. coli*
ESBL *E. coli*ESBL *K. oxytoca* ESBL *Citrobacter* sp.ESBL *M. morganii* MRSA	MDR *P. mirabilis*
ESBL *E. coli*ESBL *K. pneumoniae*	MDR non-ESBL *E. coli*
ESBL *E. coli*MRSA	MDR non-ESBL *E. coli*

*CASE* Cephalosporinase-producing; *Cefta-R* Ceftaroline-resistant; *ESBL* Extended-spectrum beta-lactamase; *MDRO* Multidrug-resistant organism; *MRSA* Methicillin-resistant *Staphylococcus aureus*

On the contrary, 29 (55.8%) episodes had a concordant identification, which were due to ESBL KP (*n* = 10), ESBL EC (*n* = 7), MRSA (*n* = 4), VIM-type carbapenemase-producing PA (n = 3), ESBL *Enterobacter cloacae* (*n* = 2), ceftaroline-resistant PA (*n* = 1), carbapenemase-producing *E. cloacae* (*n* = 1), and ceftaroline-resistant *Acinetobacter baumanii* (*n* = 1). Sites of carriage were rectal (*n* = 18), urinary (*n* = 14), respiratory (*n* = 11) and cutaneous (*n* = 7).

The global cure rate was 83/98 (84.6%).

**In univariate** analysis, there was no significant difference considering population with MDRO bacteraemia vs. non-MDRO bacteraemia, except for immunosuppression [OR 2.86; *p* = 0.0207], severity of the episode [OR 3.13; *p* = 0.0232], carriage of *Pseudomonas aeruginosa* [OR 5.24; *p* = 0.0395], and hospital-acquired infection [OR 2.49; *p* = 0.034] (Table 4).

**Table 4:** Multivariate analysis associated with multidrug-resistant organism bacteraemia

Variable	MDR bacteraemia	Univariate analysis		Multivariate analysis	
		Odds Ratio	*P* value	Odds Ratio	*P* value
Sex (male)	31/61 (50.8%)	0.70 [0.35; 1.79]	0.5683	1.04 [0.40; 2.70]	0.9403
Immunosuppression	23/33 (69.7%)	2.86 [1.17; 6.95]	*0.0207*	*2.96 [1.08; 8.13]*	*0.0354*
Severity	19/26 (73.1%)	3.13 [1.17; 8.36]	*0.0232*	2.32 [0.78; 6.88]	0.1303
Colonization MDR *Pseudomonas aeruginosa*	10/12 (83.3%)	5.24 [1.08; 25.32]	*0.0395*	2.95 [0.49; 17.77]	0.2386
Hospital-acquired	38/62 (61.3%)	2.49 [1.07; 5.78]	*0.034*	*2.62 [1.03; 6.64]*	*0.0427*

*MDR* Multidrug-resistant

italicised valued are statistically significant

**In the multivariate analysis** (Table 4), factors significantly associated with MDRO bacteraemia among colonized patient were only immunosuppression [OR = 2.96; *p* = 0.0354], and the nosocomial origin of bacteraemia [OR = 2.62; *p* = 0.0427].

## Discussion

In our study, the rate of MDRO bacteraemia among bacteraemic patients colonized with MDRO is high (53.1%).

Main factors associated with MDRO bacteraemia in those patients are immunosuppression, severity of the episode, colonization with *Pseudomonas aeruginosa,* and nosocomial infection in univariate analysis. In multivariate analysis, the only significant factors found are severity of the episode and the nosocomial origin of the infection.

The originality of our study is to focus on bacteraemic patient colonized with MDRO. Our main question is: when should we treat with probabilistic broad-spectrum antimicrobial treatment patients with known MDRO colonization and positive blood cultures?

### Risk factor for MDRO/ESBL infections

Most studies focused on colonization and infections due to multidrug-resistant *Enterobacteriaceae*, in ICU, or among immunosuppressed patients.

For example, in a 6-year prospective study, Razazi et al. screened 6303 patients admitted in ICU [22]; 843 (13.4%) had ESBL *Enterobacteriaceae* carriage detected. Among those carriers, 111 (13%) patients developed ICU-acquired pneumonia, of whom only 48 (43%) had ESBL *Enterobacteriaceae* pneumonia (6% of carriers). Moreover, considering ventilator-acquired pneumonia in ICU patients, Bruyère et al. noted in their retrospective study that the positive predictive value of digestive ESBL *Enterobacteriaceae* colonization for ESBL *Enterobacteriaceae* pneumonia was also low (41.5%) [23].

More generally, in a prospective multicenter cohort study in ICU, Barbier et al. demonstrated that ESBL *Enterobacteriaceae* infections increased carbapenem consumption, length of stay and day 28 mortality [24]. Also, ESBL *Enterobacteriaceae* infections (16.4%) were rather infrequent in carriers.

In Holland, a study focused on the predictive value of prior colonization for third-generation cephalosporin-resistant *Enterobacteriaceae* for infection due to the same microorganism [25]. This study was performed in all medical wards of an hospital, ICU included. The authors noted that, among 9422 episodes, 1657 (17.6%) of colonized patients were bacteraemic, and 64 (3.8%) were colonized with third-generation cephalosporin-resistant *Enterobacteriaceae*.

In this study, the occurrence of MDRO bacteraemia was low, corresponding to usual epidemiological data in Holland. In our study, the rate of infection due to MDRO is higher which may be due to local epidemiology.

Finally, an Israelian cohort study, with 431 carriers of carbapenem-resistant *Klebsiella pneumonia* (CRKP) included, noted that the rate of bloodstream infections (BSI) was 20% and rate of BSI due to Gram negative resistant bacteria was 80% (68/85) [26]. Among them, 19 BSI were due to CRKP and 20 to ESBL *Enterobacteriaceae*. However, in this study, no prognostic factors of CRKP BSI were identified.

The authors concluded that this raises the question regarding the use of probabilistic broad-spectrum antibiotic therapy for MDRO carriers who develop severe sepsis, as in our study.

Moreover, the authors also described frequent discordance between bacteria involved in carriage and in blood cultures [26].

Carriage of MDRO is generally the marker of high antibiotic exposure of the patient, which induces selective pressure on all flora. Yet, all MDRO are not screened, and usual screening techniques are not 100% sensitive. Therefore, a MDRO not identified during screening could be responsible for sepsis. But the indication of broad-spectrum antimicrobial treatment during sepsis among patients with MDRO carriage is still under debate, as patients do have a higher risk of MDRO infection, even if due to a different microorganism.

### Risks associated with infection due to MDRO

Regarding infection due to MRSA, colonization by MRSA is a well-known risk factor [27–29], especially in critically ill neonates children [30].

Thus, risk factors for infection due to MDRO is a complex phenomenon due to various microbiological, clinical, demographic and anamnestic characteristics [22, 23, 31, 32].

Use of algorithm to limit unnecessary use of broad-spectrum antimicrobial treatment should be encouraged [31, 32], as the one suggested by M. Basseti and J. Rodriguez Baño, which includes simple and easy to collect criteria: severity of the episode, community-acquired character, previous colonization to MDRO, indwelling device, age and previous exposure to antibiotic [33].

Lastly, new rapid diagnosis tests for bacterial resistance could help to avoid unnecessary broad-spectrum antimicrobial treatment among bacteraemic population known to be colonized by MDRO [34–37].

### Bias and weakness

The bias and weakness of our study are due to its monocentric and retrospective design, and limited sample size. Some data may be missing such as previous antimicrobial prescriptions due to memory bias. All patients were not systematically screened for MDRO at every site. Still, we studied several MDRO (ESBL bacteria, carbapenem-resistant *Enterobacteriaceae* and MRSA for example) and different sites of carriage which reflect every day practice in a tertiary care hospital. Finally, another limit of this work is that we were not able to identify patients with re-hospitalization or transferred from another hospital, which could imply an underestimation of the proportion of hospital-acquired infections.

Future research is needed to better understand the link between colonization and infection due to MDRO.

## Conclusions

According to our study, occurrence of bacteraemia due to MDRO among bacteraemic MDRO carriers was high. However, concordance between carried bacteria and blood culture bacteria was not always consistent.

Factors associated with MDRO bacteraemia were severity of the episode and nosocomial origin of the bacteraemia.

Thus, during bacteraemia among patients colonized with MDRO, if characteristics above described are present, broad-spectrum antimicrobial treatment is recommended.

## Abbreviations

BSI: bloodstream infections
CRKP: carbapenem-resistant *Klebsiella pneumonia*
EC: *Escherichia coli*
ECDC: European Centre for Disease Prevention and Control
ESBL: extended-spectrum beta-lactamase
HIV: human immunodeficiency virus
ICU: intensive care unit
KP: *Klebsiella pneumoniae*
MALDI-TOF: matrix assisted laser desorption ionisation - time of flight
MDRO: multidrug-resistant organisms
MRSA: methicillin-resistant *Staphylococcus aureus*
OR: odds ratio
PA: *Pseudomonas aeruginosa*.
